# Acute Impact of Different Reperfusion Duration Following Blood Flow Restriction on Bar Velocity during the Bench Press Exercise

**DOI:** 10.5114/jhk/194471

**Published:** 2024-12-19

**Authors:** Dawid Gawel, Robert Trybulski, Marta Bichowska-Paweska, Jakub Jarosz, Maciej Kostrzewa, Michal Wilk

**Affiliations:** 1 Institute of Sport Sciences, Jerzy Kukuczka Academy of Physical Education, Katowice, Poland.; 2 Department of Medical Sciences, Wojciech Korfanty School of Economics, Katowice, Poland.; 3 Faculty of Physical Education, Gdansk University of Physical Education and Sport, Gdansk, Poland.

**Keywords:** stiffness, athletic performance, occlusion, myotonometric assessment, ischemia

## Abstract

The main goal of this study was to evaluate the effects of different reperfusion duration following intra-conditioning blood flow restriction (BFR) on bar velocity during the bench press exercise and muscle viscoelastic properties of the triceps brachii. Eleven resistance trained males (age: 24.3 ± 4.9 years; body mass: 85.5 ± 13.2 kg; bench press 1RM: 123.6 ± 25.4 kg; training experience: 6.8 ± 5.1 years) volunteered for the study. During the experimental sessions participants performed 5 sets of 3 repetitions of the bench press exercise with a load of 60% 1RM under four different conditions: two BFR (80% AOP) and two control conditions. For the BFR conditions, cuffs were applied before each set for 4.5 min and released 30 or 60 s before the start of the set as reperfusion. Under the control conditions, BFR was not applied and the total duration of rest intervals amounted to 5 min and 5.5 min. Measurements of viscoelastic properties were conducted at baseline and immediately after completion of each set of the bench press exercise. The two-way ANOVA showed no significant condition × set interaction for mean and peak bar velocity (p = 0.93; p = 0.787; accordingly), and no main effect of condition for mean and peak bar velocity (p = 0.57; p = 0.417; accordingly). The Friedman's test showed no differences in oscillation frequency (p = 0.156), stiffness (p = 0.368), and the logarithmic decrement of tissue oscillation (p = 0.644). The results of this study indicate that BFR during rest intervals does not acutely influence mean and peak bar velocity, as well as mechanical properties of the triceps brachii regardless of the duration of reperfusion.

## Introduction

Blood flow restriction (BFR) is rapidly gaining interest among researchers and practitioners (de Mendonca et al., 2022; Wang et al., 2023). It refers to a period of circulatory restriction, typically induced by the cuffs or tourniquets, applied most proximally to the upper or lower limbs, resulting in a shortage of available oxygen in the muscle tissue (Patterson et al., 2019). To define the precise, desired level of external compression, the % of arterial occlusion pressure (AOP) is determined as the % of the individualized value of pressure at which the blood flow to a limb is ceased (Patterson et al., 2019). Various methods or modes of BFR training have been established, such as BFR used during an activity and rest periods (continuous BFR; Volga Fernandes et al., 2022; Wilk et al., 2020), only during an activity (intermittent BFR; Gepfert et al., 2021), before (pre-conditioning BFR; Bichowska-Paweska et al., 2024; da Silva Telles et al., 2020) or after an activity (post-conditioning BFR; Daab et al., 2021), as well as only during the rest periods (intra-conditioning BFR; Jarosz et al., 2021). Among the aforementioned modes of BFR, intra-conditioning BFR stands as an emergent method, fitted to remedy several unpropitious facets associated with BFR. Firstly, the cuffs are applied during rest intervals, therefore during the activity or the specific task the movement structure remains unaffected. Secondly, the use of this mode of BFR has been shown to decrease discomfort and perceptual responses in comparison to continuous BFR (Schwiete et al., 2021), which may be associated with shorter BFR duration. Reduced exposure to BFR allows not only to reduce the pain and discomfort, but also to apply higher cuff pressure (equal to or exceeding 100% AOP), which has been shown to positively influence performance under BFR (Gepfert et al., 2020; Wilk et al., 2020) and could not be applied for longer duration.

However, the amount of available studies related to intra-conditioning BFR remains scarce. Nonetheless, they partially warrant the use of this mode of BFR, especially regarding power output performance. It has been shown that the use of BFR during rest intervals allows to increase or maintain high levels of power performance (bar velocity and power output) during resistance exercise, in both the upper (Wilk et al., 2021) and lower limbs (Trybulski et al., 2022). Interestingly, it has been reported that the beneficial acute performance changes occurred only in the latter sets of exercise, but not during the initial sets (Trybulski et al., 2022; Wilk et al., 2021). On the other hand, Gawel et al. (2024) did not show a positive effect of intra-conditioning BFR on bar velocity regardless of the applied pressure. Similarly, in regard to strength-endurance performance, Trybulski et al. (2023) reported that the use of intra-conditioning BFR also did not result in an increased number of performed repetitions. However, in all of the aforementioned studies different variables related to the application of BFR, as well as different exercise modes were used. Therefore, given the conflicting results, it is suggested that these variables may influence the final acute effect of intra-conditioning BFR and concomitantly impact each other.

There is an abundance of modalities related to the methodology of using BFR e.g., the timing of BFR application, its duration, the number of BFR cycles or the pressure of the cuffs. However, as previously proposed by Trybulski et al. (2022), a significant aspect related to the effectiveness of intra-conditioning BFR might be the duration of reperfusion, which may be defined as restoration of blood flow perfusion with attendant reoxygenation of tissues following BFR (Eltzschig and Eckle, 2011). However, to this point the impact of different reperfusion duration has not been evaluated. Previous research related to resistance exercise solely utilized reperfusion duration of 30 s (Gawel et al., 2024; Trybulski et al., 2022), moreover some of the studies did not accurately report these variables (Jarosz et al., 2021; Wilk et al., 2021). To the best of our knowledge the longest duration of reperfusion reported during intra-conditioning BFR was 60 s, however these data are related to sprint performance (Fostiak et al., 2022). It might be suspected that prolonging BFR duration (>30 s) diminishes the potential performance-enhancing effect of intra-conditioning BFR, which was partially corroborated by Jarosz et al. (2023). That study showed that the application of BFR (5 min; 80%AOP) resulted in relevant alterations in mechanical properties of the rectus femoris muscle at rest. However, these changes were short-lasting and reverted to baseline during 30-s reperfusion. Moreover, it should be noted that in that study BFR was applied at rest and no training intervention was performed. Nonetheless, it seems that the role of reperfusion may be associated with muscle mechanical properties. Further, it has been suggested that fluctuations in muscle fluid may influence muscle performance (force production), as passive tension in skeletal muscle increases proportionately to fluid volume (Sleboda et al., 2019). Sleboda et al. (2019) showed that even small changes in muscle volume (5%) led to significant increases in passive force (>10%). Therefore, the monitoring of the mechanical properties of the muscles may also shed light on the mechanism inducing responses observed under BFR. However, currently the literature regarding muscle mechanical properties evaluation (myotonometry) and BFR treatment remains scarce. Further, it is still unclear, whether such an effect also occurs during exercise. Thus, the use of myotonometry may provide insight into the role of reperfusion and allow for a better understanding of how BFR may acutely influence performance.

Therefore, the prime goal of this study was to perform an assessment of the acute effects of different reperfusion duration on bar velocity changes during the bench press exercise and mechanical and contractile properties of the triceps brachii long head. Given that previous studies most often utilized reperfusion duration which lasted 30 s (Gawel et al., 2024; Trybulski et al., 2022) or less (Jarosz et al., 2021), 30 and 60 s reperfusion duration was chosen for this study. Considering the fact that increases in power performance following BFR treatment were reported when the duration of reperfusion lasted 30 s or less (Jarosz et al., 2021; Trybulski et al., 2022; Wilk et al., 2021), we hypothesized that only the shorter (30 s) reperfusion would result in increased bar velocity. Moreover, given the results of the study by Jarosz et al. (2023), it was hypothesized that viscoelastic properties of the triceps brachii long head would not be affected.

## Methods

### Participants

Eleven resistance-trained, healthy males (age: 24.3 ± 4.9 years; body mass: 85.5 ± 13.2 kg; body height: 178.5 ± 5.6 cm; bench press 1RM: 123.6 ± 25.4 kg; training experience: 6.8 ± 5.1 years) volunteered for the study. The following inclusion criteria were established: a) free from neuromuscular and musculoskeletal disorders (for at least 6 months before the start of the experiment), b) experienced in resistance training (>3 years of resistance training), c) bench press 1RM equal or exceeding 130% body mass, subsequently verified during the 1RM test, and d) free of cardiovascular diseases, including atrial fibrillation, arterial hypertension (blood pressure exceeding 140/90 mmHg), thrombosis and myocardial insufficiency (verified via self-declaration). Participants were fully briefed about the risks that may occur during the course of the study and provided their written informed consent. They were also notified about the possibility of their withdrawal from the study at any time and instructed to maintain their usual sleep and dietary habits. Further, participants were instructed not to consume any stimulants or alcohol during the experiment. An online, gratuitous generator (randomization.org) was used for randomization of the subjects. Each participant was given an individual number and sequence of their sessions. Moreover, no information about the prospective experiment outcomes was provided to participants. The research protocol was audited and then approved by the Bioethics Committee for Scientific Research at the Academy of Physical Education in Katowice, Katowice, Poland (approval code: 2/2019; approval date: 14 November 2019) and all procedures were in accordance with the ethical standards of the Declaration of Helsinki, 1983.

### Design and Procedures

**Figure 1:**
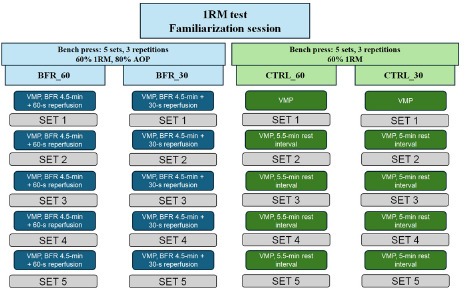
Schematic representation of the study design. BFR_60: BFR condition (4.5-min BFR + 60-s reperfusion); BFR_30: BFR condition (4.5-min BFR + 30-s reperfusion); CTRL_60: control condition (5.5-min rest interval); CTRL_30: control condition (5-min rest interval); VMP: viscoelastic muscle properties measurements; 1RM: one repetition maximum; AOP: arterial occlusion pressure

The experiment followed a randomized crossover design. In counterbalanced order each participant took part in four testing conditions (two BFR conditions and two control conditions), set three to seven days apart (Figure 1). Two weeks before the start of the main testing sessions, a familiarization session was performed, followed by a 1RM test executed one week later. During each experimental session, participants performed the bench press exercise with a load of 60% 1RM (five sets of three repetitions; Wilk et al., 2021). For BFR conditions, BFR (80% AOP) was applied during rest intervals for 4.5 min and followed by 30 s (BFR_30) or 60 s (BFR_60) reperfusion (total duration of rest intervals for BFR_30 and BFR_60 conditions amounted to 5 and 5.5 min, respectively). For control conditions, BFR was not applied and the rest intervals lasted 5 min (CTRL_30) and 5.5 min (CTRL_60). Measurements of viscoelastic properties (oscillation frequency [Hz], muscle stiffness [N/m] and the logarithmic decrement of tissue oscillation [relative arbitrary unit characterizing the dampening of tissue oscillation]) of the triceps brachii long head were conducted via a handheld myotonometer at baseline and immediately after completion of each set of the bench press exercise. A linear position transducer was used to measure the values of peak and mean bar velocity during the bench press exercise. All testing sessions took place in the Strength and Power Laboratory at the Jerzy Kukuczka Academy of Physical Education in Katowice, Katowice, Poland. During the course of the experiment, no changes in the procedure were made.

### Familiarization Session

Two weeks before the main experiment, participants attended the familiarization session. During the familiarization session, measurements of frequency, decrement and stiffness were performed at baseline and after every single set of the bench press exercise at the initial determination middle point on the triceps brachii long head, which was marked so that in subsequent sessions an independent person verified the correctness of the measurement point. After performing a general warm-up according to the participant’s training habits, each participant performed three sets of three repetitions of the bench press exercise (60% of estimated 1RM). BFR (60% AOP) was used before the first set and during the rest intervals (5 min) between sets (Wilk et al., 2021). The goal of the familiarization session was to decrease the possibility of learning effects during the main sessions.

### 1RM Strength Test

A free-weight bench press 1RM test was performed one week before the main experiment. At first, during the warm-up participants pedaled on a cycle-ergometer for 5 min (heart rate of ~130 bpm), and then proceeded to preferred dynamic stretching, consistent with their usual routine (~8 min). Then, participants performed the bench press exercise using 20, 40, 60 and 80% of their estimated 1RM for 12, 10, 5, and 3 repetitions, respectively. Afterwards, the load was subsequently increased for each following attempt (2.5 to 10 kg), and the rest interval lasted 5 min (Filip-Stachnik et al., 2021). This process was repeated until failure, however, a maximum of five attempts was allowed. The bench press technique was supervised by three certified and experienced strength and conditioning experts. Hand placement on the barbell was volitional, yet participants were instructed to replicate it during each set. Participants were given an instruction to perform the eccentric phase of the bench press with a volitional movement tempo and the concentric phase of the movement with maximal velocity. All repetitions were performed without raising the hips off the bench, intentionally pausing the movement or bouncing the barbell of the chest (Trybulski et al., 2022a). Eleiko Olympic barbell (2.8-cm diameter, 1.92-m length; International, Halmstad, Sweden) was used for all testing sessions performed during the experiment.

### Experimental Sessions

Towards a better equation of the results, the experimental procedure was based on previous studies related to intra-conditioning BFR (Gawel et al., 2024; Wilk et al., 2021). For every condition during the main testing sessions each participant performed five sets of three repetitions of the bench press exercise (60% 1RM). The movement tempo in the eccentric and concentric phases of the movement was maximal. BFR was not applied under the control conditions, however, during other experimental sessions BFR was administered prior to the beginning of the first set and during every rest interval in between. During BFR conditions the pressure of the cuffs was determined as ~80% AOP. The rest interval between sets amounted to 5 min (BFR_30, CTRL_30) and 5.5 min (BFR_60, CTRL_60). A linear position transducer (Tendo Power Analyzer, Tendo Sport Machines, Trencin, Slovakia) was utilized to measure the values of peak and mean bar velocity. Linear position transducers have been shown to exhibit good reliability for the measurement of bar velocity (intra-class correlation co-efficient [ICC] of 0.81 for peak bar velocity, ICC of 0.83 for mean bar velocity) during the free-weight bench press exercise at 60% 1RM (Moreno-Villanueva et al., 2021; Orange et al., 2020). Peak bar velocity was acquired from the best repetition performed in each set, while mean bar velocity was obtained from three repetitions performed in each set. Mechanical properties of the triceps brachii long head were measured via a hand-held myotonometer (MyotonPRO, Myoton AS, Tallinn, Estonia) at baseline and immediately after completion of each set of the bench press exercise. For measurements of oscillation frequency, stiffness and decrement, MyotonPRO has been shown to be a reliable and valid device (ICC [0.83–0.99]; Bailey et al., 2013; Bizzini et al., 2003).

### Blood Flow Restriction Procedure

Seven-cm wide cuffs (Fit Cuffs ApS, Denmark) were utilized for the experiment. For both BFR conditions, cuffs were applied as high as possible on both limbs, in proximity to the axillary fossa. For the BFR conditions (BFR_30, BFR_60), before each set BFR was administered for 4.5 min and released 30 s (BFR_30) or 60 s (BFR_60) before the beginning of a set (4.5-min BFR + 30-s reperfusion or 4.5-min BFR + 60-s reperfusion, respectively). After the completion of the warm-up and a 5-min rest interval, the value of full arterial occlusion pressure (100% AOP) was determined for each participant via a Doppler device with a 2-mHz probe and an OLED screen (Edan Instruments, Shenzhen, China; Wilk et al., 2021). For each limb, the measurement was performed twice (participants remained seated). The average value obtained from the two measurements (the differences were within 20 mmHg) was used to establish the pressure of the cuffs for the exercise protocol (Wilk et al., 2021). For all BFR conditions, cuff pressure was determined as ~80% AOP (127 ± 16 mmHg).

### Measurement of Muscle Mechanical Properties

Assessment of contractile and mechanical characteristics of the triceps brachii long head was performed with a hand-held, portable myotonometer with a triaxial accelerometer MyotonPRO (Myoton AS, Tallinn, Estonia; Ce et al., 2020). The following properties were measured: oscillation frequency (Hz), muscle stiffness (N/m), and the logarithmic decrement of tissue oscillation (relative arbitrary unit). The MyotonPRO triaxial accelerometer was set at 3200 Hz (Trybulski et al., 2022a) with an average value acquired from three consecutive measurements (mechanical force of 0.4 N for 15 ms). All measurements were conducted on both limbs with participants lying prone, with the arms at their sides and forearms pronated. Each measurement was conducted initially on the left upper limb, and immediately after on the right upper limb at baseline and immediately after each set of the bench press exercise. As a measuring point, the middle part of the muscle belly was chosen and marked with a marker in order to allow for precise replication of the positioning for the following measurements (Chuang et al., 2012). The same professional was responsible for locating the measurement point for all participants.

### Statistical Analysis

IBM SPSS Statistics for Macintosh, Version 25.0 was used for the analysis of the data (IBM Corp., Armonk, N.Y., USA). All data were shown as means with standard deviations (± SD) and their 95% confidence intervals (CIs). Statistical significance was set at *p* < 0.05. The Shapiro-Wilk test was utilized in order to verify the normality of data distribution and the Mauchly’s test was employed to verify assumption of variance sphericity. The Friedman rank tests were used to investigate the influence of BFR on muscle mechanical properties. Moreover, two-way ANOVAs (4 conditions × 5 sets) were used to examine the impact of BFR on barbell velocity during the bench press exercise. When a significant interaction or main effect was found, the post-hoc tests with Bonferroni correction were used to analyze the pairwise comparisons. Further, standardized effect size (ES) was utilized to express the magnitude of mean differences (thresholds for qualitative descriptors of Hedges g; large >0.80, medium 0.21–0.79, small ≤0.20).

## Results

### Muscle Mechanical Properties

The Shapiro-Wilk test showed a significant violation of data distribution in all muscle mechanical properties.

The Friedman's test did not reveal any differences in oscillation frequency (test = 56.727; *p* = 0.156; Kendall’s W = 0.101), stiffness (test = 49.665; *p* = 0.368; Kendall’s W = 0.088), and the logarithmic decrement of tissue oscillation (test = 42.872; *p* = 0.644; Kendall’s W = 0.076) (Table 1).

**Table 1:** Comparison of muscle mechanical properties.

Group		Pre (95%CI)	Set 1 (95%CI)	Set 2 (95%CI)	Set 3 (95%CI)	Set 4 (95%CI)	Set 5 (95%CI)
**Stiffness [N/m]**							
CTRL_30	L	275.2 ± 40.1 (251.5 to 198.8)	272.1 ± 45.5 (245.2 to 299)	268.7 ± 45.8 (241.6 to 295.8)	262.8 ± 36 (241.6 to 284.1)	273.3 ± 62 (236.6 to 309.9)	263 ± 39.3 (239.8 to 286.2)
	R	273.6 ± 38.5 (250.8 to 296.3)	273.7 ± 44.3 (247.5 to 299.8)	276.5 ± 47.7 (248.3 to 304.7)	267.8 ± 32.8 (248.4 to 287.2)	275.6 ± 39.8 (252.1 to 299.1)	274.7 ± 36 (253.4 to 295.9)
CTRL_60	L	268.4 ± 32.7 (249.1 to 287.7)	273 ± 36.2 (251.6 to 294.4)	269.7 ± 31.5 (251.1 to 288.3)	268.7 ± 32.5 (249.5 to 287.9)	273.8 ± 33.3 (254.1 to 293.5)	261.3 ± 31.1 (242.9 to 279.6)
	R	271.4 ± 36.4 (249.9 to 292.9)	269.1 ± 38.6 (246.2 to 291.9)	267.7 ± 35.5 (246.7 to 288.6)	270.6 ± 42 (245.7 to 295.4)	268.4 ± 33.5 (248.6 to 288.2)	266.9 ± 35.8 (245.7 to 288.1)
BFR_30	L	286.7 ± 46.5 (259.2 to 314.1)	295.6 ± 78.4 (249.3 to 341.9)	283 ± 48.7 (254.2 to 311.8)	301.8 ± 61.3 (265.6 to 338.1)	297 ± 55.9 (263.9 to 330.1)	279.8 ± 52.9 (248.5 to 311.0)
	P	290.5 ± 34.6 (270.0 to 311.0)	263.9 ± 36.4 (242.4 to 285.4)	275.4 ± 28.8 (258.4 to 292.4)	280.6 ± 26.8 (264.8 to 296.4)	291.1 ± 43 (265.7 to 316.5)	308.9 ± 56.4 (275.6 to 342.2)
BFR_60	L	276.2 ± 32.3 (257.1 to 295.2)	275.8 ± 40.2 (252.1 to 299.6)	284.8 ± 46.4 (257.3 to 312.2)	287.9 ± 48.8 (259.1 to 316.7)	278.9 ± 60.3 (243.3 to 314.6)	288.3 ± 49 (259.4 to 317.3)
	R	273.4 ± 35.6 (252.4 to 294.4)	279.8 ± 35.5 (258.9 to 300.8)	276.8 ± 26.1 (261.3 to 292.2)	279.2 ± 35.3 (258.3 to 300.0)	279.2 ± 36.4 (257.9 to 300.9)	289.8 ± 42.8 (264.5 to 315.1)
	**Oscillation Frequency [Hz]**						
CTRL_30	L	16.5 ± 1.7 (15.4 to 17.5)	16.3 ± 2.3 (14.9 to 17.6)	16 ± 2.1 (14.8 to 17.2)	15.8 ± 2 (14.7 to 17.2)	16.6 ± 3.2 (14.7 to 18.2)	16 ± 1.5 (15 to 16.9)
	R	16.2 ± 1.7 (15.2 to 17.2)	16.2 ± 2 (15 to 17.4)	16.2 ± 2.2 (14.9 to 17.5)	16 ± 1.4 (15.2 to 16.9)	16.2 ± 1.7 (15.2 to 17.3)	16 ± 1.9 (14.9 to 17.1)
CTRL_60	L	16 ± 1.5 (15.2 to 16.9)	16.3 ± 1.5 (15.4 to 17.2)	16 ± 1.4 (15.2 to 16.9)	16.1 ± 1.5 (15.2 to 17)	16.2 ± 1.6 (15.3 to 17.2)	15.8 ± 1.5 (15 to 16.7)
	R	16.1 ± 1.6 (15.1 to 17.1)	16.1 ± 1.3 (15.3 to 16.9)	16 ± 1.5 (15.1 to 16.9)	16.2 ± 1.8 (15.1 to 17.3)	15.9 ± 1.5 (15 to 16.8)	15.8 ± 1.7 (14.8 to 16.8)
BFR_30	L	16.9 ± 1.9 (15.7 to 18)	17.4 ± 3 (15.6 to 19.1)	16.9 ± 2.3 (15.5 to 18.3)	17.6 ± 2.7 (16.1 to 19.2)	17.3 ± 2.1 (16.1 to 18.5)	16.8 ± 2.1 (15.6 to 18)
	R	16.6 ± 1.4 (15.8 to 17.4)	15.7 ± 1.7 (14.7 to 16.7)	16.3 ± 1.4 (15.5 to 17.1)	16.6 ± 1 (16 to 17.2)	16.8 ± 1.8 (15.7 to 17.9)	17.4 ± 2.1 (16.2 to 18.7)
BFR_60	L	16.4 ± 1.8 (15.4 to 17.4)	16.5 ± 2.1 (15.3 to 17. 7)	16.6 ± 1.9 (15.5 to 17. 7)	16.9 ± 2.2 (15.6 to 18.2)	16.2 ± 2.3 (14.8 to 17.6)	16.7 ± 2 (15.5 to 17.9)
	R	16.1 ± 1.4 (15.3 to 16.9)	16.6 ± 1.5 (15.7 to 17.4)	16.4 ± 1.3 (15.6 to 17.1)	16.4 ± 1.5 (15.5 to 17.3)	16.5 ± 1.8 (15.4 to 17.5)	16.8 ± 1.8 (15.8 to 17.9)
	**Logarithmic Decrement of Tissue Oscillation [relative arbitrary unit]**						
CTRL_30	L	0.98 ± 0.17 (0.88 to 1.08)	1 ± 0.18 (0.89 to 1.11)	1.01 ± 0.13 (0.93 to 1.08)	1.03 ± 0.19 (0.92 to 1.15)	1.05 ± 0.18 (0.94 to 1.15)	1.03 ± 0.11 (0.96 to 1.10)
	P	1.03 ± 0.15 (0.94 to 1.12)	0.99 ± 0.11 (0.93 to 1.05)	1.03 ± 0.11 (0.97 to 1.10)	1.01 ± 0.11 (0.94 to 1.07)	1.02 ± 0.14 (0.94 to 1.10)	1.02 ± 0.14 (0.95 to 1.08)
CTRL_60	L	1.03 ± 0.14 (0.94 to 1.11)	1.03 ± 0.14 (0.94 to 1.11)	1.01 ± 0.15 (0.92 to 1.10)	1.03 ± 0.15 (0.94 to 1.12)	1.02 ± 0.13 (0.94 to 1.09)	1.07 ± 0.13 (0.99 to 1.14)
	R	0.99 ± 0.13 (0.92 to 1.07)	1.01 ± 0.12 (0.94 to 1.08)	1.01 ± 0.15 (0.92 to 1.09)	1.07 ± 0.28 (0.90 to 1.23)	1.06 ± 0.12 (0.99 to 1.13)	1 ± 0.11 (0.94 to 1.06)
BFR_30	L	1.06 ± 0.15 (0.97 to 1.15)	0.99 ± 0.14 (0.91 to 1.07)	1.02 ± 0.17 (0.92 to 1.12)	1.03 ± 0.2 (0.91 to 1.15)	0.92 ± 0.11 (0.85 to 0.99)	0.99 ± 0.09 (0.94 to 1.05)
	R	1.02 ± 0.09 (0.97 to 1.08)	0.97 ± 0.22 (0.93 to 1.01)	0.98 ± 0.08 (0.93 to 1.03)	0.96 ± 0.07 (0.91 to 1.00)	0.99 ± 0.1 (0.93 to 1.05)	1 ± 0.15 (0.91 to 1.09)
BFR_60	L	1.01 ± 0.17 (0.91 to 1.11)	1.03 ± 0.22 (0.90 to 1.15)	1.02 ± 0.15 (0.93 to 1.11)	1.07 ± 0.22 (0.94 to 1.20)	1.03 ± 0.12 (0.96 to 1.10)	1.05 ± 0.21 (0.93 to 1.17)
	R	1 ± 0.17 (0.90 to 1.09)	0.98 ± 0.13 (0.90 to 1.05	0.98 ± 0.15 (0.89 to 1.08)	0.95 ± 0.15 (0.86 to 1.04)	0.94 ± 0.14 (0.86 to 1.02)	0.98 ± 0.16 (0.88 to 1.08)

All data are presented as mean with standard deviation while 95% confidence intervals (CI) are presented in parentheses. CTRL_30: control condition (5-min rest interval); CTRL_60: control condition (5.5-min rest interval); BFR_30: BFR condition (4.5 BFR + 30-s reperfusion); BFR_60: BFR condition (4.5 BFR + 60-s reperfusion); Pre: measurement at baseline; L: left upper limb; R: right upper limb

### Barbell Velocity

**Figure 2:**
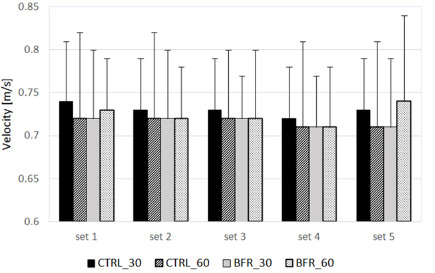
Mean bar velocity (m/s) during each set of the bench press exercise under experimental conditions. CTRL_30: control condition (5-min rest interval); CTRL_60: control condition (5.5-min rest interval); BFR_30: BFR condition (4.5-min BFR + 30-s reperfusion); BFR_60: BFR condition (4.5-min BFR + 60-s reperfusion)

Two-way ANOVA revealed no significant interaction (F = 0.468; *p =* 0.93; η^2^ = 0.041), nor the main effect of condition (F = 0.563; *p* = 0.57; η^2^ = 0.049) for mean bar velocity (Figure 2; Table 2).

**Table 2:** Mean and peak values of bar velocity.

Group	Set 1 (95%CI)	Set 2 (95%CI)	Set 3 (95%CI)	Set 4 (95%CI)	Set 5 (95%CI)
**Mean bar velocity [m/s]**					
CTRL_30	0.74 ± 0.07 (0.70 to 0.78)	0.73 ± 0.06 (0.70 to 0.77)	0.73 ± 0.06 (0.70 to 0.77)	0.72 ± 0.06 (0.69 to 0.76)	0.73 ± 0.6 (0.70 to 0.77)
CTRL_60	0.72 ± 0.1 (0.71 to 0.73)	0.72 ± 0.1 (0.71 to 0.73)	0.72 ± 0.08 (0.67 to 0.77)	0.71 ± 0.1 (0.70 to 0.72)	0.71 ± 0.1 (0.70 to 0.72)
BFR_30	0.72 ± 0.08 (0.67 to 0.77)	0.72 ± 0.08 (0.67 to 0.77)	0.72 ± 0.05 (0.69 to 0.75)	0.71 ± 0.06 (0.67 to 0.75)	0.71 ± 0.08 (0.66 to 0.76)
BFR_60	0.73 ± 0.06 (0.69 to 0.77)	0.72 ± 0.06 (0.68 to 0.76)	0.72 ± 0.08 (0.67 to 0.77)	0.71 ± 0.07 (0.67 to 0.75)	0.74 ± 0.1 (0.73 to 0.75)
**Peak bar velocity [m/s]**					
CTRL_30	0.95 ± 0.12 (0.88 to 1.02)	0.96 ± 0.09 (0.91 to 1.01)	0.94 ± 0.1 (0.93 to 0.95)	0.92 ± 0.1 (0.91 to 0.93)	0.92 ± 0.1 (0.91 to 0.93)
CTRL_60	0.95 ± 0.16 (0.86 to 1.04)	0.96 ± 0.13 (0.88 to 1.04)	0.96 ± 0.12 (0.89 to 1.03)	0.93 ± 0.14 (0.85 to 1.01)	0.93 ± 0.14 (0.85 to 1.01)
BFR_30	0.94 ± 0.11 (0.87 to 1.01)	0.93 ± 0.11 (0.86 to 1)	0.93 ± 0.09 (0.88 to 0.98)	0.93 ± 0.09 (0.88 to 0.98)	0.92 ± 0.1 (0.91 to 0.93)
BFR_60	0.99 ± 0.16 (0.9 to 1.08)	0.97 ± 0.16 (0.88 to 1.06)	0.97 ± 0.15 (0.88 to 1.06)	0.96 ± 0.11 (0.89 to 1.03)	0.96 ± 0.11 (0.89 to 1.03)

All data are presented as mean with standard deviation while 95% confidence intervals (CI) are presented in parentheses. CTRL_30: control condition (5-min rest interval); CTRL_60: control condition (5.5-min rest interval); BFR_30: BFR condition (4.5 BFR + 30-s reperfusion); BFR_60: BFR condition (4.5 BFR + 60-s reperfusion)

**Figure 3:**
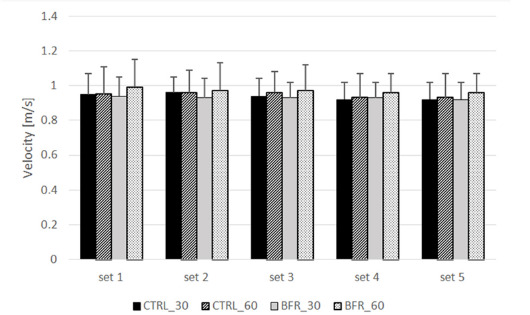
Peak bar velocity (m/s) during each set of the bench press exercise under experimental conditions. CTRL_30: control condition (5-min rest interval); CTRL_60: control condition (5.5-min rest interval); BFR_30: BFR condition (4.5-min BFR + 30-s reperfusion); BFR_60: BFR condition (4.5-min BFR + 60-s reperfusion)

Two-way ANOVA revealed no significant interaction (F = 0.659; *p* = 0.787; η^2^ = 0.057), nor the main effect of condition (F = 0.973; *p* = 0.417; η^2^ = 0.081) for peak bar velocity (Figure 3; Table 2).

The comparison of ES among the experimental conditions is presented in Table 3.

**Table 3:** Differences in effect size between experimental conditions.

Comparison	Set 1		Set 2		Set 3		Set 4		Set 5	
	MV	PV	MV	PV	MV	PV	MV	PV	MV	PV
CTRL_30 vs. CTRL_60	0.23	0	0.12	0	0.14	0.23	0.12	0.10	0.24	0.10
CTRL_30 vs. BFR_30	0.27	0.09	0.14	0.3	0.18	0.16	0.14	0.16	0.28	0
CTRL_30 vs. BFR_60	0.15	0.28	0.17	0.08	0.14	0.28	0.24	0.51	0.12	0.51
CTRL_60 vs. BFR_30	0	0.07	0	0.25	0.15	0.28	0	0	0	0.08
CTRL_60 vs. BFR_60	0.12	0.25	0	0.07	0.13	0.07	0	0.24	0.3	0.23
BFR_30 vs. BFR_60	0	0.36	0	0.29	0	0.32	0	0.3	0.33	0.37

MV: mean bar velocity; PV: peak bar velocity; CTRL_30: control condition (5-min rest interval); CTRL_60: control condition (5.5-min rest interval); BFR_30: BFR condition (4.5 BFR + 30-s reperfusion); BFR_60: BFR condition (4.5 BFR + 60-s reperfusion)

## Discussion

The purpose of this study was to determine the impact of the reperfusion duration following BFR applied during rest intervals between sets on acute power performance changes and muscle viscoelastic properties (i.e., stiffness, oscillation frequency, and the logarithmic decrement of tissue oscillation) of the triceps brachii long head measured via myotonometry. The primary result of this study was the lack of significant increments in power performance (mean and peak bar velocity during the multiple-set bench press exercise at 60% 1RM) regardless of the reperfusion duration, which was in opposition to our hypothesis. However, although it might be concluded that the reperfusion duration following BFR did not acutely influence power performance changes, it should be noted that there were no increases in bar velocity under every condition, thus it still remains infeasible to assess the role of reperfusion during intra-conditioning BFR.

Further, no relevant differences in muscle viscoelastic properties were recorded, partially warranting our hypothesis. Nonetheless, muscle viscoelastic properties also did not change under every condition.

The duration of reperfusion following BFR has been suggested as a possible factor impacting its effectiveness (Gawel et al., 2024; Jarosz et al., 2023; Trybulski et al., 2022). Husmann et al. (2018) reported a diminished effect of BFR on muscle fatigue (impairment in muscle contractile function) after 2 min of reperfusion. However, that study was related to continuous BFR applied during isometric low-load resistance exercise. On the other hand, a different study by Jarosz et al. (2023) indicated that 30 s of reperfusion may be sufficient to diminish fatigue associated with the application of BFR. Jarosz et al. (2023) showed that the application of BFR (5 min; 80%AOP) did lead to relevant alterations to mechanical properties of the rectus femoris muscle at rest, however, during 30- s reperfusion these values returned to baseline. Although the aforementioned findings may provide insight into muscle fatigue development following BFR and its impact on both performance (Husmann et al., 2018) as well as muscle properties (Jarosz et al., 2023), they are not related to intra-conditioning BFR. Therefore, regarding intra-conditioning BFR, the duration of reperfusion might be one of the distinct factors differentiating among available study protocols (Jarosz et al., 2021; Trybulski et al., 2022; Wilk et al., 2021). The improvements in power performance (significant improvement in bar velocity and power output) occurred when the set of resistance exercise was performed immediately after the release of the cuffs (Jarosz et al., 2021; Wilk et al., 2021). However, 30-s reperfusion has also been reported to be beneficial to sustain high-level performance in the face of accumulating fatigue (Trybulski et al., 2022). Further, the present study is the first to apply 60-s reperfusion during multi-set resistance exercise. However, taking into consideration that the absence of relevant increases in bar velocity occurred under every condition, it is still unclear whether shorter or longer reperfusion duration would be more beneficial. Moreover, it is possible that other factors subsequently discussed may be of greater importance compared to reperfusion duration in regard to the effectiveness of intra-conditioning BFR in improving power performance.

Given the potential role of reperfusion in enhancing power performance, its duration may indeed be significant, however, another issue to consider is its relationship with other factors such as the duration of BFR. Foremost results in regard to enhanced power performance occurred when the duration of BFR was ~5 min (Trybulski et al., 2022; Wilk et al., 2021) or less (~2.5 min; Jarosz et al., 2021). Moreover, according to Ghosh et al. (2000), in order to reach the threshold for an ischemic stimulus in humans 4 min of BFR is adequate duration. These findings are partially corroborated by Gawel et al. (2024) who showed that 6.5-min BFR (5 sets of 3 repetitions; 60% 1RM; 30-s reperfusion) did not increase bar velocity during the bench press exercise, regardless of the applied pressure (50% AOP, 80% AOP, 20 mmHg). Therefore, it is likely that 6.5-min BFR exceeds the maximal threshold to influence positive changes in power performance. Furthermore, the influence of the cuffs pressure on the effectiveness of BFR cannot be dismissed. However, it might be concluded that lengthening the duration of BFR does not lead to superior results. On the other hand, the present study utilized shorter BFR duration (~4.5 min) and also did not show increases in performance under every condition, partially contradicting previous research (Trybulski et al., 2022; Wilk et al., 2021). However, it should be noted that due to distinct reperfusion duration (30 s or 60 s) the total duration of the rest interval and reperfusion differed (5 min and 5.5 min, respectively). The effectiveness of BFR may be influenced by various, associated factors. The available data indicate ~5 min or less to be the most effective BFR duration to improve power performance (Trybulski et al., 2022; Wilk et al., 2021). Thus, the duration of BFR, the duration of reperfusion and the total sum or the ratio of these duration times might play a role in regard to the effectiveness of intra-conditioning BFR, however, due to the paucity of data further investigations are required.

Interestingly, Salagas et al. (2022) showed that a single cycle of BFR, used only before the first set (4-set protocol), caused significant increases in bar velocity during the bench press exercise at 60% 1RM during sets 1–3, however, not during the fourth set. It should be noted that Salagas et al. (2022) utilized higher cuff pressure (100% AOP) compared to the aforementioned research (Gawel et al., 2024; Jarosz et al., 2021; Trybulski et al., 2022; Wilk et al., 2021); furthermore, not only BFR, but also reperfusion lasted 5 min. The study by Salagas et al. (2022) demonstrated positive effects of BFR on power performance already in the first set, contrary to previous studies by Wilk et al. (2021) and Trybulski et al. (2022), thus indicating that the amount of BFR cycles and cuff pressure may be also significant factors related to its effectiveness. Therefore, given the available body of research, such an intervention (a single cycle of BFR; 100% AOP) is insufficient to induce changes in the latter sets, which is contrary to the other studies where BFR (multiple cycles of BFR; 60–80% AOP) applied during rest intervals influenced performance in the latter, but not during the initial sets (Trybulski et al., 2022; Wilk et al., 2021). Furthermore, although Gawel et al. (2024) found no differences regarding power performance when different cuff pressure (50% AOP, 80% AOP, 20 mmHg) was applied, currently there is no available study related to intra-conditioning BFR which utilized a pressure of 100% AOP. In the present study, 80% AOP was used, which may be one of the factors (insufficient pressure) that contributed to the lack of increases in power performance. Therefore, it is suggested that along with the % of AOP, the duration of reperfusion and the duration of BFR may be of significance, however, it needs to be highlighted that the magnitude of their impact is concurrently influenced by other variables, e.g., the number of BFR cycles or the method of applying BFR (Salagas et al., 2022; Trybulski et al., 2022; Wilk et al., 2021).

In the present study, no differences in muscle viscoelastic properties (i.e., stiffness, oscillation frequency, and the logarithmic decrement of tissue oscillation) occurred, which is in line with our hypothesis. Changes in stiffness or muscle tone most often have corresponded with a decrease in performance and accumulation of fatigue (Klich et al., 2020; Wang, 2016). Wojdala and Krzysztofik (2023) showed augmented muscle stiffness of the triceps brachii post-exercise in comparison to pre-exercise measures after performing the bench press exercise with the maximal number of repetitions (70% 1RM). Furthermore, Trybulski et al. (2022a) reported a trend for augmented stiffness of the triceps brachii and decreased bar velocity during the bench press exercise (70% 1RM) performed to failure. Thus, previous research has shown increases in muscle stiffness, simultaneously with progressive fatigue after a resistance exercise protocol. A physiological factor related to the alterations to the mechanical properties of the muscles is an increase in intramuscular pressure, accompanied by a higher level of intracellular fluid (Friden et al., 1986; Jarosz et al., 2023; Krzysztofik et al., 2023). Moreover, it affects muscle metabolism, tissue oxygenation and delays muscle recovery and function due to an impaired blood flow (Jessee et al., 2018; Kablan et al., 2021; Krzysztofik et al., 2023). Therefore, the overall performance is decreased. It has been shown that changes in muscle volume are associated with increases in passive force (Sleboda et al., 2019), thus the monitoring of muscle viscoelastic properties may contribute to exploring the influence of BFR on power performance. However, the available data related to this matter remain scarce. To the best of our knowledge, there is only one study related to this issue, yet it refers to passive BFR (Jarosz et al., 2023). Jarosz et al. (2023) indicated that 30-s reperfusion might be sufficient to diminish fatigue associated with the application of BFR, and allow for a decrease in intramuscular pressure (after 30 s of reperfusion mechanical properties of the muscle reverted to baseline). It should be noted, however, that in that study BFR was applied at rest, therefore it cannot be accurately compared to the present study. Nonetheless, the absence of changes in muscle viscoelastic properties in the present study might indicate that the application of BFR during rest intervals followed by reperfusion may be beneficial to reducing fatigue (absence of relevant changes in bar velocity in the consecutive sets) during the training protocol. However, it should be taken into account that muscle viscoelastic properties remained unchanged under every condition, therefore it still remains infeasible to draw definite conclusions. Nevertheless, monitoring muscle viscoelastic properties may be beneficial in future research regarding intra-conditioning BFR.

The present study is not without limitations which should be addressed. Firstly, BFR has been previously shown to induce positive effects on endocrine and metabolic responses (Caru et al., 2019; Teixeira et al., 2018). Additionally, Torma et al. (2021) reported that intra-conditioning BFR may influence mitochondrial biogenesis and the gene expression of angiogenesis, thus, as a result, influence muscle hypertrophy. However, in the present study physiological assessment was not performed. Therefore, although no acute responses were recorded following BFR, the possible occurrence of chronic adaptations should also be considered. Secondly, during the bench press, which is a multi-joint exercise (Tsoukos et al., 2024), the assessment of viscoelastic properties was performed solely on one muscle (the triceps brachii long head), thus the obtained results may be different for other muscles involved. Moreover, it should be mentioned that in order to assess two distinct BFR duration times (30 s vs. 60 s), the total duration of the rest interval (4.5 min) and reperfusion amounted to two distinct duration times (5 min and 5.5 min, respectively).

## Conclusions

It can be concluded that the application of intra-conditioning BFR does not acutely influence mean and peak bar velocity, regardless of the duration of reperfusion (30 s vs. 60 s). Moreover, the absence of changes in mechanical and contractile properties of the triceps brachii long head were recorded after each set under every condition. However, it should be noted that there were no increases in bar velocity under every condition, thus the role of reperfusion in intra-conditioning BFR warrants further investigation. Moreover, the effectiveness of BFR is most likely influenced by various factors, including the % of AOP, the duration of BFR, the number of BFR cycles and the duration of reperfusion concurrently impacting each other. Despite the growing body of research related to this topic, further research is needed to formulate definitive conclusions and recommendations for practice and research.
